# Accuracy of Deep Learning Algorithms for the Diagnosis of Retinopathy of Prematurity by Fundus Images: A Systematic Review and Meta-Analysis

**DOI:** 10.1155/2021/8883946

**Published:** 2021-08-06

**Authors:** Jingjing Zhang, Yangyang Liu, Toshiharu Mitsuhashi, Toshihiko Matsuo

**Affiliations:** ^1^Department of Regenerative and Reconstructive Medicine (Ophthalmology), Graduate School of Interdisciplinary Science and Engineering in Health Systems, Okayama University, Okayama 7008530, Japan; ^2^Department of Epidemiology, Graduate School of Medicine, Dentistry and Pharmaceutical Sciences, Okayama University, Okayama 7008558, Japan; ^3^Center for Innovative Clinical Medicine, Okayama University Hospital, Okayama University, Okayama 7008558, Japan

## Abstract

**Background:**

Retinopathy of prematurity (ROP) occurs in preterm infants and may contribute to blindness. Deep learning (DL) models have been used for ophthalmologic diagnoses. We performed a systematic review and meta-analysis of published evidence to summarize and evaluate the diagnostic accuracy of DL algorithms for ROP by fundus images.

**Methods:**

We searched PubMed, EMBASE, Web of Science, and Institute of Electrical and Electronics Engineers Xplore Digital Library on June 13, 2021, for studies using a DL algorithm to distinguish individuals with ROP of different grades, which provided accuracy measurements. The pooled sensitivity and specificity values and the area under the curve (AUC) of summary receiver operating characteristics curves (SROC) summarized overall test performance. The performances in validation and test datasets were assessed together and separately. Subgroup analyses were conducted between the definition and grades of ROP. Threshold and nonthreshold effects were tested to assess biases and evaluate accuracy factors associated with DL models.

**Results:**

Nine studies with fifteen classifiers were included in our meta-analysis. A total of 521,586 objects were applied to DL models. For combined validation and test datasets in each study, the pooled sensitivity and specificity were 0.953 (95% confidence intervals (CI): 0.946–0.959) and 0.975 (0.973–0.977), respectively, and the AUC was 0.984 (0.978–0.989). For the validation dataset and test dataset, the AUC was 0.977 (0.968–0.986) and 0.987 (0.982–0.992), respectively. In the subgroup analysis of ROP vs. normal and differentiation of two ROP grades, the AUC was 0.990 (0.944–0.994) and 0.982 (0.964–0.999), respectively.

**Conclusions:**

Our study shows that DL models can play an essential role in detecting and grading ROP with high sensitivity, specificity, and repeatability. The application of a DL-based automated system may improve ROP screening and diagnosis in the future.

## 1. Introduction

Retinopathy of prematurity (ROP) occurs in preterm infants on supplemental oxygen, which helps to improve survival but may contribute to blindness [1]. The ROP grades are complicated and include aggressive ROP (AP-ROP), prethreshold ROP, or regression of ROP, as well as stages, zones, extent, and preplus/plus diseases of ROP [1, 2]. ROP can be diagnosed by binocular direct or indirect ophthalmoscopy [3], and fundus photographs are taken by digital retinal cameras, such as RetCam. Due to difficulties associated with eye contact photography in newborns and limited technological expertise of ophthalmologists, as well as financial and ethical issues, ROP screening is not a common practice. However, early treatment can improve the structural and functional outcomes [4]. Therefore, developing a screening method for early diagnosis of ROP is essential.

Artificial intelligence has the potential to revolutionize the current disease diagnosis pattern in ophthalmology and generate a significant clinical impact [5]. Deep learning (DL), a technology of machine learning (ML), was introduced to artificial neural networks in 2000 [6]. DL can automatically grade images and has been applied to ophthalmology for signal processing and diagnostic retinal imaging [5, 7]. The deep convolutional neural network/convolutional neural network (DCNN/CNN) is a DL technique that is widely used to interpret medical images through classifiers. It is a multilayer structure comprising a convolutional layer, a nonlinear processing unit, and a subsampling layer [8]. DL must be trained with high mathematical precision but can be implemented using a lower precision computer; thus, the automatic detection system can be applied in a general hospital.

In recent years, DL algorithms have been widely applied to ophthalmology for the diagnosis of various diseases, such as diabetic retinopathy (DR), age-related macular degeneration (AMD), and ROP [5, 9]. However, due to the lack of a public database for ROP and hence the difficulty in obtaining a large clinical dataset, the development of DL algorithms for diagnosing ROP lags behind other retinal diseases. Some studies have used DL models to process retinal images by vessel segmentation or zone identification and have recommended the application of the feature-based images for further clinical diagnosis or DL model building [10–12]. Some studies followed this flow to build an entire DL model for diagnosing ROP. They applied the model to extract features relevant to ROP, such as tortuosity and dilation of vessel, and applied these images to the classifiers of the DL model [13]. Other studies have suggested that using original retinal images to build a DL model without limited features may reserve more information [13, 14]. Some accuracy measurements, such as accuracy, sensitivity, and specificity, were calculated to evaluate the performance of DL algorithms in detecting ROP using fundus images compared to the clinical methods.

The validation dataset is used to tune the model's hyperparameters, whereas the test dataset provides an unbiased evaluation of a final model fit. Most studies have only verified the DL model through internal validation using a test dataset derived from the training dataset, rather than external validation that uses an independent test dataset. We typically diagnose diseases using a plethora of diagnostic methods, but DL limits evidence to images. The DL model is complex and a “black-box” that the mechanism is unknown. These reasons make the diagnostic results unstable and unreliable, hindering their use in clinical practice. Therefore, we conducted a meta-analysis to summarize and compare the published evidence to evaluate the accuracy of DL algorithms for the diagnosis of ROP by fundus images.

## 2. Methods

### 2.1. Systematic Review

We followed the Preferred Reporting Items for Systematic Reviews and Meta-Analyses (PRISMA) guidelines, which are based on Cochrane's Handbook for Systematic Reviews, to conduct and report on the current study [15, 16]. For our study, we searched the following public databases: PubMed, EMBASE, Web of Science, and Institute of Electrical and Electronics Engineers Xplore Digital Library (IEEE). We systematically searched using the most appropriate free-text terms (“Retinopathy of Prematurity (MeSH),” OR “Prematurity Retinopathy”, OR “Retrolental Fibroplasia”) AND (“Deep learning (MeSH),” OR “Hierarchical learning,” OR “Convolutional Neural Network,” OR “Deep Neural Network”) to find relevant articles published between January 1, 2000, and June 13, 2021. In addition, the relevant articles were tracked through automatic e-mail alerts from the databases during the preparation of our manuscript.

### 2.2. Inclusion and Exclusion Criteria

Two authors (Zhang and Liu) independently screened the titles and abstracts for retrieved articles to be considered for the systematic review. We selected the articles from the initial screening and retained them for full-text review, excluding editorials, letters to the editor, reviews, commentary, policy, contribution, conference, book, and articles with traditional methods for detecting ROP. All the included studies had to fulfill the following criteria: (1) the studies were restricted to peer-reviewed articles published in English (conference abstracts or proceedings were excluded), (2) the studies provided information on the dataset, diagnosis, and grading criteria of ROP, and the number of research object (e.g., images, cases (eyes), or infants) in each group, (3) the studies described the DL algorithms for distinguishing ROP using a binary classifier and provided an evaluation, such as accuracy, sensitivity, and specificity, using the area under the receiver operating characteristics curve (AUC, AUROC), or precision and recall, using the area under the precision-recall curve (AUPR). All the studies meeting the inclusion criteria at this stage were additionally reviewed by the same two authors to ensure they were appropriate for the final analysis. Disagreements were resolved by discussion with another expert (Matsuo).

### 2.3. Data Extraction and Quality Assessment

Two researchers (Zhang and Liu) individually retrieved all information from the selected articles and extracted the following items: author, publication year, dataset characteristics, definition and grade of ROP, DL model, training, validation and test set characteristics, and all accuracy values of validation and testing. True-positive (TP), true-negative (TN), false-positive (FP), and false-negative (FN) values were calculated for a meta-analysis. If the TP/TN/FP/FN values were not quantitatively expressed or could not be calculated from the composition of dataset and accuracy measurements, the study was excluded.

Unlike ordinary diagnostic accuracy studies, there are no generally accepted and appropriate quality criteria for evaluating the accuracy of DL algorithms. We referred to the Quality Assessment of Diagnostic Accuracy Studies-2 (QUADAS-2) [17] and PLASTER (a framework for evaluating DL performance) [18] to select some assessment factors, such as image selection and preprocessing, clear description of DL algorithms and algorithm evaluation, reference standards to label images for the classifier of the CNN, and flow and timing of ROP. Inconsistent results between authors in data extraction and quality assessment were resolved through discussion.

### 2.4. Statistical Analysis

A meta-analysis was conducted using Meta-analysis of diagnostic and screening tests (Meta-DiSc, Version: 1.4, Universidad Complutense, Barcelona, Spain) [19]. An overall test performance was evaluated by separately combining TP/TN/FP/FN values of the validation and test datasets in each study. Separate subgroup analyses were performed: the validation and test dataset, separately and the definition of ROP and the grade of ROP, separately. The DerSimonian–Laird random-effects model was applied to the pooled data. The descriptive forest plot for pooled sensitivity and specificity values, positive and negative likelihood ratio (PLR/NLR), diagnostic odds ratios (DOR), and the AUC of summary receiver operating characteristics curves (SROC) [20] were used to summarize overall test performance. Statistical significance was expressed with 95% confidence intervals (CIs). The AUC criteria are 0.90–1 (excellence), 0.80–0.90 (good), 0.70–0.80 (fair), 0.60–0.70 (poor), and 0.50–0.60 (failure).

Threshold effect and nonthreshold effect testing were used to examine heterogeneity, assess potential biases, and evaluate the accuracy factors. The threshold effect exists when different cutoffs or thresholds are used to define a positive (or negative) test result in different studies [19]. The nonthreshold effect may exist in case of chance and variations in the study population, index test, reference standard, study design, and conducted partial verification bias [21]. For DL models, the nonthreshold effect may be caused by image segmentation methods, feature extraction methods, and classifiers. A Spearman correlation coefficient (*r*) between the logit of true-positive rate (TPR) and logit of false-positive rate (FPR, 1-specificity) was calculated, and a strong positive correlation *r* with a *p* value less than 0.05 suggests the threshold effect [18]. If there was a threshold effect, the included studies might have used different thresholds to define positive and negative results; therefore, the sensitivity and specificity values were heterogeneous, and the pooled results should be referred to with caution [22]. The nonthreshold effect test, using the chi-square value of pooled accuracy estimate and Cochran-*Q* value of DOR, was implemented. If the heterogeneity was beyond the specifications, the test results would have a low *p* value [19, 23].

## 3. Results

### 3.1. Selected Studies and General Characteristics

**Figure 1:**
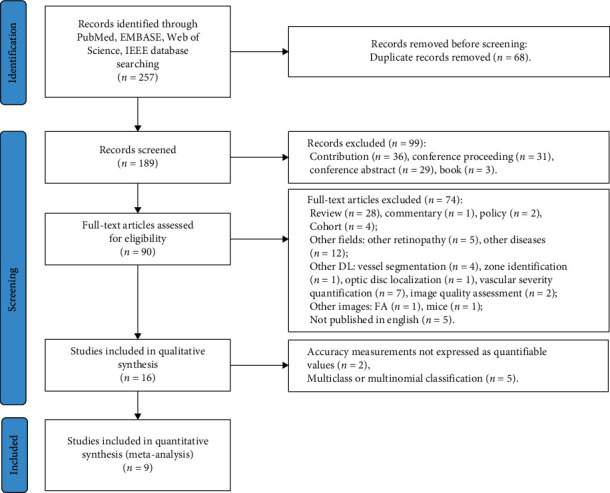
Prisma flow diagram for study selection.

Figure 1 shows details of the screening stage.  Table 1 provides the nine studies with fifteen classifiers, which were included in our systematic review by meta-analysis.

Publication years ranged from 2018 to 2021. All training datasets were hospital-based rather than built as a database after quality control. A total of 521,586 objects were applied to DL models. All the included studies, except one study, reported the type of digital camera used to obtain the retinal images [33]. Nevertheless, since all the data were collected after the improvement of the neonatal fundus image quality [14], we retained this study and ruled out the potential bias due to the image quality [34]. The diagnosis of ROP and its grade is credible due to the use of similar reference standard and the consistent label by professionals. All the included studies developed DL models to detect ROP from normal subjects, and five studies further distinguished two ROP grades [24–26, 28, 30]. Most studies evaluated the algorithm using *k*-fold cross-validation or by developing several modules and then selecting the best one to apply to the final DL model. Considering that not all the studies reported complete accurate measurements, we only applied the dataset with available TP/TN/FP/FN values to meta-analysis. The accuracy of the validation and testing datasets were 0.785–0.99 and 0.856–0.988, respectively.

### 3.2. Meta-Analysis

**Figure 2:**
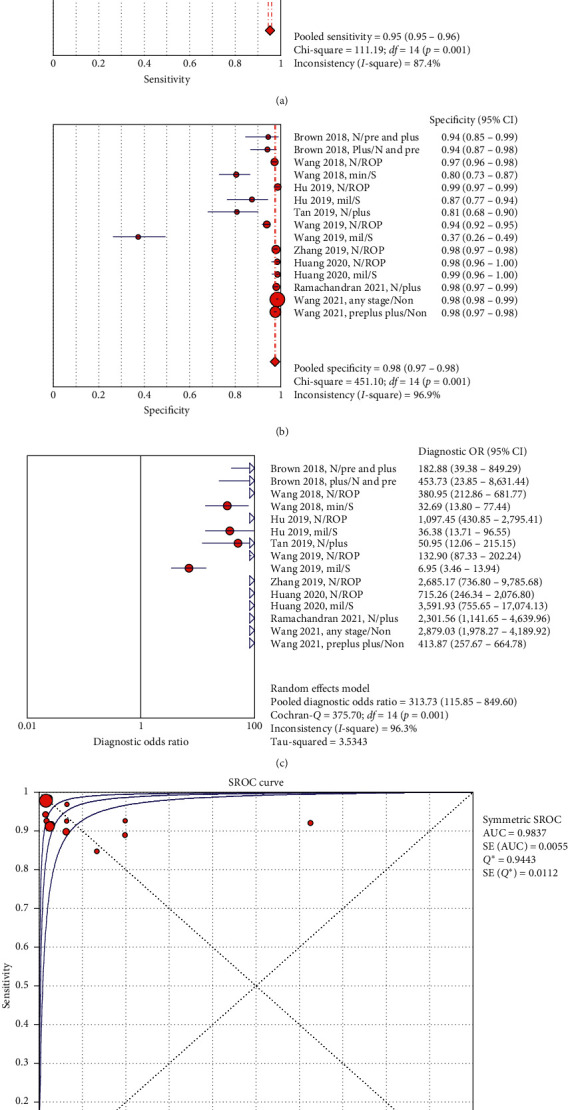
Performance of the DL models for detecting and grading ROP in primary analyses. Forest plots of sensitivities (a), specificities (b), and diagnostic odds ratios (DOR) (c), with respective confidence intervals, respectively, as well as to assess the heterogeneity in accuracy estimates across studies. Plots of individual study results in ROC space with receiver operating characteristics curve for all classifiers included (SROC) (d).

In the threshold effect test for primary analyses, Spearman correlation coefficient (*r*) was −0.561 (*p*=0.030), suggesting the absence of a threshold effect. In the subgroup analyses, none of the subgroups obtained any significant *r* to show evidence of the threshold effect (all *p* values > 0.05).  Table 2 provides the results of primary and subgroup analyses.  Figure 2 shows the performance of the DL models in detecting and grading ROP in the primary analyses. In the pooling of sensitivity, specificity, PLR, NLR, and DOR, the nonthreshold effect tests showed high heterogeneity from the nonthreshold effect across all studies and all subgroup analyses (all the chi-square and Cochran-*Q* with *p* values of <0.01).

We explored the heterogeneous source of included studies according to the primary and subgroup analyses, quality assessment, and testing results of threshold and nonthreshold effects. There was no evidence of the threshold effect, possibly because the “threshold” has different meanings in clinical diagnosis and DL models. For DL models, positive (or negative) is defined based on probability rather than a certain decision; therefore, different DL models may share the same threshold of probability. According to the results of the nonthreshold effect test and quality assessment, there may be a risk beyond bias for random reasons. Since all the images were from infants and were obtained using RetCam, the bias of patient selection could be avoided. Additionally, the images were labeled according to a standard reference or consistent opinion; however, they were obtained by different ophthalmologists and processed by various technologies, creating a risk of bias for object (images) selection. In addition, as the images were acquired from different quadrants of the fundus, there is a possibility of misclassification of the ROP grades. Considering that the flow and timing of disease were part of the classification criteria, the risk of bias among studies cannot be avoided. For DL models, different dataset composition, CNN structure, and hyperparameter setting could also cause heterogeneity.

## 4. Discussion

### 4.1. Principal Findings

This systematic review and meta-analysis evaluated the performance of DL algorithms in detecting and grading ROP using RetCam images compared to the clinical methods. The results showed that DL models have a promising performance in ROP screening, and their diagnosis has clinical relevance with the ophthalmologist's. The main results are that DL algorithms (DCNN/CNN) achieved high sensitivity and specificity in identifying ROP and distinguishing two grades of ROP; the PLR, NLR, and DOR values indicate good test performance. All the accuracy values based on AUC are over 0.97, which is classified as high when above 0.9 [35]. Therefore, the included studies suggested that the DL-based automatic diagnosis system for ROP was effective. Comparing primary and subgroup analyses, the specificity, PLR, NLR, and DOR of distinguishing ROP grades are less satisfactory but matches expectations, possibly because ROP progression is a continuous spectrum and the definition of positive and negative is unclear. The primary and subgroup analyses had nonthreshold effects, creating considerable uncertainty around accuracy estimates in this meta-analysis; however, the AUC obtained from the SROC curve is quite robust to heterogeneity [36].

Some issues should be considered when using DL models for ROP diagnosis. (1) Early diagnosis and screening are essential for ROP, and most studies focus on higher sensitivity rather than specificity when selecting cut-offs to build DL models. However, in clinical practice, the low specificity may impede its adaption due to various considerations, including financial implications [37]. (2) When the membership of the test set is not balanced (e.g., the actual negative is far more than the actual positive), the specificity may not reflect the variety of true negative. Therefore, it is better to use precision with the precision-recall (P-R) curve to evaluate accuracy. In our studies, only two studies reported the P-R curve [28, 38]. (3) The performance of the DL model is affected by the image quality and disease manifestation [37, 39]. Therefore, the clinical performance of proposed DL models requires more external verification to avoid overestimation due to overfitting and bias [24, 25, 35]. (4) The ROP course is continuous, meaning that both binary and multiple classifications of images are crude in clinical practice. (5) The ROC curve can only evaluate binary classification, and although some studies developed DL models with multiclass or multinomial classification to diagnose ROP, they could not be included in the meta-analysis. (6) Contrary to the diagnosis of DR and AMD, ROP imaging mostly requires pupil dilation to avoid quality issues due to nonmydriatic fundus photography [40]. This could explain why the accuracy of ROP diagnosis is better than that of DR (the pooled AUC was 0.97) [9]. (7) Additionally, contrary to DR, the gold standard, fluorescein fundus angiography, is rarely applied to ROP diagnosis in infants. Subsequently, the vessel segmentation techniques may play a more critical role in the automatic diagnosis system. However, studies that have independently developed vessel segmentation techniques to label features are limited [41]. (8) Most studies did not evaluate the diagnosis of late-stage ROP because their DL models are based on retinal blood vessel morphology, which is difficult to visualize in late ROP.

### 4.2. Opportunities and Challenges

There are immense opportunities for applying DL algorithms to develop the automatic ROP identification system based on the fundus images. Implementation of automated systems based on DL algorithms would improve the efficiency and coverage of ROP screening and subsequently promote early treatment to reduce retinal detachment and loss of vision. However, several challenges need to be addressed. For a given DL model, the specificity increases while sensitivity decreases; thus, further studies should improve the algorithms to overcome the difficulties of raising both indexes. The DL models trained by a given dataset are specific to that dataset, and generalization of the DL model is unreliable. The DL algorithm is considered a “black-box.” Although some studies limited some features to make the process open, the inflexible learning method rather than experience hinders ophthalmologists from accepting the diagnosis by the DL model. The DL algorithm is isolated, whereas ophthalmologists have an integrated knowledge system; this may affect the patients' trust in the diagnosis. Most DL models are optimized for classification rather than diagnosis. Notably, ROP diagnosis comprises identifying, grading, defining affected zones, and identifying symptoms of preplus or plus disease, which may not be possible using DL models. Besides, the DL model could not make differential diagnosis of ROP from other retinal diseases, such as retinal vascular dysplasia. Additionally, the quality of images used to train the CNN model affects diagnostic accuracy. High-quality images increase DL power consumption; thus, it is necessary to maximize energy efficiency [18, 34]. Notably, DL tends to overfit. Most training sets of DL models for DR can involve approximately 10,000 images, but this number of images is insufficient for ROP. Some studies expanded the dataset by image augmentation, but none of the CNN models for ROP was regularized to prevent overfitting [42]. Most fundus images are taken by ophthalmologists, who deliberately focus on some abnormal regions for precise diagnosis and grading of ROP. In addition, ophthalmologists rely on the patients' clinical history, such as oxygen supplementation, for accurate diagnosis. In contrast, the CNN model may not perform well for images with subtle findings that most ophthalmologists cannot identify. The ImageNet-trained CNNs are biased towards texture rather than shape, which is different from human observers [43]. In identifying DR or retinal hemorrhage [44], the focus is on changes to texture, but for ROP, diagnosis is based on alterations of the shape of vascular tissues. This may explain why the diagnostic performance of pretrained ImageNet for ROP is less satisfactory.

### 4.3. Strengths and Limitations

Our study is the most comprehensive systematic review and meta-analysis to evaluate the performance of the DL model to detect and grade ROP. However, our study has several limitations. First, we could not reduce the heterogeneity from the nonthreshold effect among the studies as this difference is inherent to the imaging mode and internal features of the DL model. However, it does not affect the value of this study in providing an overview of the diagnostic accuracy of DL models. Second, accuracy measurements for some studies or some subdatasets were unavailable to us. Third, we could only evaluate the performance of DL models using the accuracy measurements provided by individual studies rather than calculating the accuracies by directly applying the images to the DL models in practice. Fourth, due to the varied DL arithmetic logics, it was difficult to conduct a subgroup analysis based on the models to assess the bias. Fifth, we only systematically analyzed the DL models with binary classifiers. Since multiple classifiers yielded a probabilistic interpretation representing each classification, the distribution of these probability outputs could be illustrated in the violin plot but could not be pooled. Sixth, some studies neither validated algorithms on external data nor compared algorithm performance against other professionals; thus, the generalization of DL algorithms could not be assessed [37]. Seventh, the objects of DL models could be infants, cases (eyes), or images, and an infant or a case may contain several images. We could not estimate the effect because the classification based on several images might be more accurate, and a small sample size might affect the diagnostic accuracy [45].

## 5. Conclusions

Our study findings show that DL models can play an essential role in detecting and grading ROP with high sensitivity, specificity, and repeatability. The application of a DL-based automated system may change the approach to ROP screening and diagnosis in the future, which may improve healthcare. Earlier detection and timely treatment might halt disease progression at an earlier stage and prevent the onset of complications.

## Figures and Tables

**Table 1:** Characteristics of nine studies for the systematic review and meta-analysis.

General characteristics	Dataset characteristics		Definition and grade of ROP		
Author	Year, data source	Camera	Reference standard	Dataset	Identification and grade						

Brown et al. [24]	2018, i-ROP	RetCam	RSD, images and clinical diagnosis	5511 images	4535 N, 805 pre and 172 plus						
Wang et al. [25]	2018, hospital and web	RetCam 3	ICROP, CRYO-ROP, and ETROP	3722 cases	2823 N and 899 ROP; 382 Min and 295 S						
Hu et al. [26]	2019, hospital	RetCam 3	Consistent label	2668 images	1484 N and 1184 ROP; 382 Mil and 295 S						
Tan et al. [27]	2019, ART-ROP	RetCam	Images and clinical diagnosis	6974 images	5336 N and 1638 plus						
Wang et al. [28]	2019, hospital	NR	Consistent label	11000 images	7559 N and 3441 ROP; 529 Mil and 1204 S						
Zhang et al. [29]	2019, hospital	RetCam 2/3	The same criteria	19543 images	11298 N and 8245 ROP						
Huang et al. [30]	2020, hospital	RetCam	ICROP + consistent label	18808 images	1222 N and 1129 ROP; 1189 Mil and 1174 S						
Ramachandran et al. [31]	2021, KIDROP	RetCam 3	Consistent label	289 infants	200 N and 89 plus						
Wang et al. [32]	2021, hospital	RetCam 2/3	Consistent label	52249 images	6363 any stage and 42177 N; 885 pre or plus and 17223 N						

DL model characteristics												
Author	Neural network				Algorithm evaluation		Classification					

Brown et al. [24]	CNN: U-Net and Inception V1				The 5-fold cross-validation		N/pre and plus					
							Plus/N and pre					
Wang et al. [25]	DNN: Id-Net and Gr-Net				NR		N/ROP					
							Min/S					
Hu et al. [26]	CNN: a pretrained ImageNet (VGG16, inception V2, and ResNet-50)				Select the best module and image size		N/ROP					
							Mil/S					
Tan et al. [27]	CNN: Inception V3				NR		N/plus					
Wang et al. [28]	CNN: a pretrained ImageNet (Inception V2, Inception V3, and ResNet-50)				Select the best module		N/ROP					
							Mil/S					
Zhang et al. [29]	DNN: AlexNet, VGG16, and GoogLeNet				Select the best module		N/ROP					
Huang et al. [30]	DNN: VGG16, VGG19, MobileNet, InceptionV3, and DenseNet				Select the best module and then 5-fold cross-validation		N/ROP					
							Mil/S					
Ramachandran et al. [31]	CNN: a pretrained ImageNet (Darknet-53 network)				Select the best module		N/plus					
Wang et al. [32]	CNN: ResNet18, DenseNet121, and EfficientNetB2				Five independent classifiers validation		Preplus plus/non					
							Any stage/non					
Accuracy values												
Author	Negative vs. positive	TD	VD	ACC	SN	SP	AUC	TED	ACC	SN	SP	AUC

Brown et al. [24]	N vs. pre and plus	80%	20%	NR	NR	NR	0.94	100 (from the same set with TD)	0.91	0.93	0.94	NR
	N and pre vs. plus	80%	20%	NR	NR	NR	0.98			1	0.94	NR
Wang et al. [25]	N vs. ROP	2226	298	NR	0.9664	0.9933	0.9949	944 (from web)	NR	0.8491	0.9690	NR
	Min vs. S	2004	104	NR	0.8846	0.9231	0.9508	106 (from web)	NR	0.933	0.736	NR
Hu et al. [26]	N vs. ROP	2068	300	0.97	0.96	0.98	0.9922	406 (from the same set with TD)	NR	0.900	0.989	NR
	Mil vs. S	466	100	0.84	0.82	0.86	0.9212	31 (from ROP in TED)	NR	0.944	0.923	NR
Tan et al. [27]	N vs. plus	5579	1395	0.973	0.966	0.98	0.993	90 (external set)	0.856	0.939	0.807	NR
Wang et al. [28]	N vs. ROP	8507	1228	0.927	0.8999	NR	NR	1265 (from TD)	NR	NR	NR	NR
	Mil vs. S	1175	269	0.785	0.9235	NR	NR	289 (from ROP in TED)	NR	NR	NR	NR
Zhang et al. [29]	N vs. ROP	17801	1742	0.988	0.935	0.995	0.998	1742 (from the same set with TD)	0.988	0.935	0.995	0.998
Huang et al. [30]	N vs. ROP	2351	368 cases	NR	Average 0.911	Average 0.992	NR	101 (from the same set with TD)	0.96	0.966	0.952	0.97
	Mil vs. S	2363	339 cases	NR	Average 0.987	Average 0.985	NR	85 (from ROP in TED)	0.988	1	0.984	0.99
Ramachandran et al. [31]	N vs. plus	About 80%	About 20%	0.99	0.99	0.98	0.9947	1610 (from the same set with TD)	NR	0.98	0.98	NR
Wang et al. [32]	Non vs. any stage	36235	4813	NR	0.972	0.984	0.9977	7492 (from the same set with TD)	NR	0.982	0.985	0.9981
	Non vs. preplus and plus	13524	1866	NR	0.909	0.984	0.9882	2718 (from the same set with TD)	NR	0.918	0.97	0.9827

ROP, retinopathy of prematurity. *Reference Standard*. Based on images: RSD, a reference standard diagnosis; ICROP, International Classification of ROP, and based on both images and clinical information: CRYO-ROP, Cryotherapy for Retinopathy of Prematurity; ETROP, early treatment ROP; N, normal, pre, preplus disease; plus, plus disease; Min, minor; Mil, mild; S, severe; i-ROP, Imaging and Informatics in Retinopathy of Prematurity; ART-ROP, Auckland Regional Telemedicine ROP image library; KIDROP, Karnataka Internet assisted diagnosis of ROP program; DL, deep learning; CNN, convolutional neural network; DNN, deep neural network; DCNN, deep convolutional neural network; TD, training dataset; VD, validation dataset; TED, test dataset. Total data set includes TD, VD, and TED; ACC, accuracy; SN, sensitivity; SP, specificity; AUC, area under the receiver operating curve; NR, not reported.

**Table 2:** The results of primary and subgroup analyses.

	Sensitivity (95% CI)	Specificity (95% CI)	PLR (95% CI)	NLR (95% CI)	DOR (95% CI)	AUC (95% CI)	Spearman *r* (*p* value)
Primary analyses	0.953 (0.946–0.959)	0.975 (0.973–0.977)	19.265 (8.431–44.019)	0.065 (0.040–0.105)	313.73 (115.85–849.60)	0.984 (0.978–0.989)	−0.561 (0.030)
Validation dataset	0.934 (0.922–0.945)	0.973 (0.969–0.977)	26.232 (6.978–98.616)	0.076 (0.046–0.125)	359.58 (94.565–1367.3)	0.977 (0.968–0.986)	−0.612 (0.060)
Test dataset	0.969 (0.961–0.975)	0.977 (0.974–0.979)	22.853 (12.593–41.475)	0.049 (0.026–0.092)	522.92 (213.89–1278.4)	0.987 (0.982–0.992)	−0.280 (0.354)
Define ROP	0.956 (0.949–0.962)	0.979 (0.977–0.981)	30.118 (19.225–47.184)	0.055 (0.033–0.092)	576.21 (238.54–1391.9)	0.9895 (0.9849–0.9941)	−0.503 (0.138)
Distinguish ROP	0.931 (0.906–0.952)	0.856 (0.826–0.882)	7.927 (2.049–30.674)	0.097 (0.038–0.252)	88.655 (13.251–593.13)	0.9820 (0.9641–0.9999)	−0.600 (0.285)

*Note.* PLR, positive likelihood ratio; NLR, negative likelihood ratio; DOR, diagnostic odds ratios.

## Data Availability

The data supporting this meta-analysis are from previously reported studies and datasets, which have been cited. The processed data are available from the corresponding author upon request.
